# The Bacterial Symbiont *Wolbachia* Induces Resistance to RNA Viral Infections in Drosophila melanogaster

**DOI:** 10.1371/journal.pbio.1000002

**Published:** 2008-12-23

**Authors:** Luís Teixeira, Álvaro Ferreira, Michael Ashburner

**Affiliations:** Department of Genetics, University of Cambridge, Cambridge, United Kingdom; University of Lausanne, Switzerland

## Abstract

*Wolbachia* are vertically transmitted, obligatory intracellular bacteria that infect a great number of species of arthropods and nematodes. In insects, they are mainly known for disrupting the reproductive biology of their hosts in order to increase their transmission through the female germline. In Drosophila melanogaster, however, a strong and consistent effect of *Wolbachia* infection has not been found. Here we report that a bacterial infection renders *D. melanogaster* more resistant to *Drosophila* C virus, reducing the load of viruses in infected flies. We identify these resistance-inducing bacteria as *Wolbachia*. Furthermore, we show that *Wolbachia* also increases resistance of *Drosophila* to two other RNA virus infections (Nora virus and Flock House virus) but not to a DNA virus infection (Insect Iridescent Virus 6). These results identify a new major factor regulating D. melanogaster resistance to infection by RNA viruses and contribute to the idea that the response of a host to a particular pathogen also depends on its interactions with other microorganisms. This is also, to our knowledge, the first report of a strong beneficial effect of *Wolbachia* infection in D. melanogaster. The induced resistance to natural viral pathogens may explain *Wolbachia* prevalence in natural populations and represents a novel *Wolbachia*–host interaction.

## Author Summary

Many symbiotic bacteria confer fitness benefits to the organisms that they infect. *Wolbachia* are one of the most widespread intracellular bacteria, infecting a great number of species of insects. Here we show that in the fruit fly Drosophila melanogaster, infection with *Wolbachia* increases resistance to a natural pathogen of *Drosophila*, an RNA virus called *Drosophila* C virus. Furthermore, we show that *Wolbachia* also increases resistance of *Drosophila* to two other RNA viruses (Nora and Flock House virus) but not to a DNA virus (Insect Iridescent Virus 6). These results identify a significant new factor that regulates D. melanogaster resistance to infection by RNA viruses. Our results add to a growing body of literature showing that the response of an organism to a particular pathogen is modulated by prior or contemporaneous interactions with other microorganisms. That the fruit fly clearly benefits from increased resistance to viruses may provide a solution to the longstanding puzzle as to why *Wolbachia* is so common in natural populations of D. melanogaster.

## Abstract 3

*Wolbachia* are one of the most widespread intracellular bacteria. In*Drosophila melanogaster*, the presence of*Wolbachia* increases resistance to infection by RNA viruses.

## Introduction

*Wolbachia* are obligatory, intracellular α-proteobacteria that infect a wide range of arthropods and filarial nematodes. They are found in 17% to 76% of surveyed arthropods and have recently been estimated to be present in 66% of all arthropod species [1–3]; therefore *Wolbachia* are one of the most widespread intracellular bacteria. Although the phylogenies of *Wolbachia* strains and their arthropod hosts show horizontal transmission of the bacteria on an evolutionary time-scale [4], these endosymbionts are mainly transmitted maternally. Consequently, *Wolbachia* strains and the species they infect form long-term associations.

*Wolbachia* were first discovered infecting the mosquito Culex pipiens in 1924 [5], but interest in these bacteria mainly arose when it was shown that infected mosquito males do not successfully breed with noninfected females [6]. This phenomenon is termed cytoplasmic incompatibility (CI) and has, since then, been found in many other insect species infected with *Wolbachia* [7]. In some hosts, *Wolbachia* can also cause feminization, male killing, or parthenogenesis [7]. All these mechanisms profoundly alter the reproductive biology of their hosts and are thought to increase the success of bacterial transmission through the female germline. In the majority of known cases, *Wolbachia* behave like reproductive parasites of their hosts.

Interestingly, in the parasitic wasp Asobara tabida, a *Wolbachia* strain is required for the inhibition of apoptosis in the germline and, consequently, normal oogenesis [8,9]. Similarly, *Wolbachia* is required for normal development and fertility in many filarial nematodes [10–13]. In all these cases, the endosymbionts are obligatory mutualists—they are essential for the survival of their host species. Curiously, examples of *Wolbachia* infections that are facultative and provide a fitness benefit are rare (e.g., [14,15]). One would, however, expect them to be frequent, since these are long-term symbioses, and *Wolbachia* fitness ultimately depends on the host fitness.

The model organism Drosophila melanogaster can also be infected with *Wolbachia*. In fact, they are detected in a large proportion of flies of natural populations and laboratory stocks [16–18]. Interestingly the presence of *w*Mel, the *Wolbachia* strain associated with D. melanogaster, does not seem to cause a strong phenotype. *w*MelPop, a *Wolbachia* variant from a laboratory stock, does causes tissue degeneration and significantly shortens the lifespan of its carriers [19]. The appearance of this strain may be an artifact of conditions in which laboratory stocks are kept, because no *w*Mel variant from natural populations with these characteristics has been discovered. *Wolbachia* also rescues the sterility of *Sex-lethal* hypomorphic mutants [20]. However, it is not known how this translates to the interaction of *Wolbachia* with wild-type flies. How natural variants of *Wolbachia* affect wild-type D. melanogaster has been extensively addressed. *w*Mel only induces a weak and transient CI phenotype in D. melanogaster [21–23], although some *Wolbachia* strains induce strong CI in the closely related D. simulans [24]. This low CI cannot explain how *Wolbachia* spreads and is maintained in wild-type populations, especially considering that, in the wild, infection is not vertically transmitted with 100% fidelity [25]. A strong hypothesis to explain *Wolbachia* presence in natural populations is that *Wolbachia* gives a fitness benefit to D. melanogaster [26,27]. Several studies have either been unable to find differences in fitness parameters or found only slight beneficial or detrimental effects of *Wolbachia* infection [25,27–31]. Moreover, even when effects were observed, they were dependent on the *Wolbachia* variant or the fly's genetic background. A clear strong beneficial effect of *Wolbachia* infection in D. melanogaster has still not been shown, and it remains a puzzle why these bacteria are so prevalent in natural populations.

D. melanogaster is a valuable tool in the study of resistance to pathogens, with many components of innate immunity signaling pathways conserved between *Drosophila* and mammals [32]. The epitome of its utility was the discovery of the involvement of *Toll*-like receptors in innate immunity. *Toll* was first discovered to be important in the resistance of *Drosophila* to fungi [33], later *Toll*-like receptors were shown to have fundamental functions in mammalian innate immunity [34]. Moreover, *Toll*-like receptors are important in the activation and modulation of mammalian adaptive immunity.

The responses of *Drosophila* to systemic infection by fungi and bacteria are increasingly well known [35]. Although less extensively, *Drosophila* has also been used as a model system to study resistance to viruses. Recent research has shown conservation between flies and mammals in their immune response to viruses. Mutations in *hopscotch*, the gene that encodes the kinase of the JAK-STAT pathway, reduce resistance to *Drosophila* C virus (*D*CV) infection and increases viral titers [36]. Interestingly, in mammals, JAK-STAT pathways are involved in cytokine signaling, including anti-viral type I interferon [37]. Work in *Drosophila* has also been important in showing that RNA interference is involved in anti-viral resistance in animals. Flies mutant in genes that encode components of this pathway, *Dicer-2*, *Argonaute-2*, and *r2d2*, are more sensitive to infection by several RNA viruses and have higher titers of viruses than the wild type [38–41].

To identify new genes involved in *Drosophila* resistance to viruses, we initiated a screen for *D*CV-sensitive flies. In doing so, we found that flies infected with intracellular bacteria were much more resistance to *D*CV infection than those that were uninfected. We identified these bacteria as *Wolbachia* and show that *D*CV titers are much lower in *Wolbachia-*infected flies. Moreover, resistance to infection extends to two other RNA viruses but not to a DNA virus. These results identify a new major factor involved in *Drosophila* resistance to RNA viruses, and the first strong beneficial effect associated with *Wolbachia* infection in D. melanogaster.

## Results

### Tetracycline Treatment Reduces Resistance to *D*CV

In order to identify new genes involved in D. melanogaster resistance to viruses, we are conducting a genetic screen for virus-sensitive mutants (LT, AF, MA, unpublished data). We have generated a collection of mutant lines by *P*-element insertional mutagenesis using the set of *w^1118^ iso* isogenic lines described in Ryder et al., 2004 [42]. We chose an isogenic background to minimize variability in the response to viral infection and, for the same reason, cleaned the initial set of lines of potential chronic viral infections using the protocol described in Brun and Plus, 1978 [43]. We test the resistance of each insertion line to *D*CV infection. *D*CV is a small, non-enveloped virus with a single-stranded, positive-sense RNA genome that belongs to the Dicistroviridae family, an insect specific family of viruses very similar to picornaviruses [44]. This virus is a natural pathogen of D. melanogaster, it is sequenced and relatively well characterized, and its infection has an easily scored lethal phenotype [43,44].

**Figure 1:**
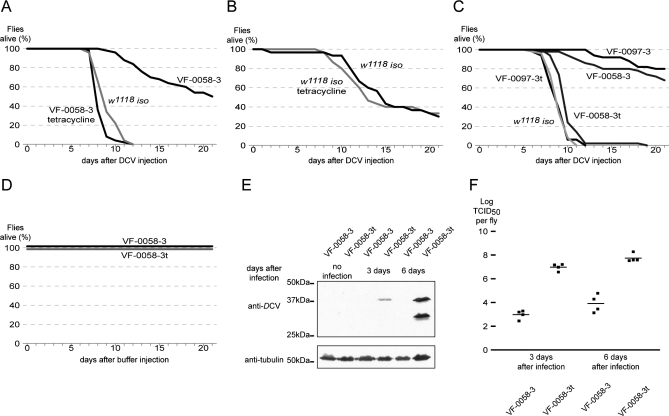
Tetracycline Treatment Increases Flies Sensitivity to *D*CV (A, B, and C) Fifty 3–6-d-old males, per sample, were injected with *D*CV, and their survival was followed daily. (A) Flies *w^1118^ iso*, VF-0058–3, and VF-0058–3 raised on tetracycline for one generation were injected with 500 TCID_50_ *D*CV. (B) Flies *w^1118^ iso* and *w^1118^* iso raised on tetracycline were injected with 50 TCID_50_ *D*CV. (C) Flies *w^1118^ iso*, VF-0058–3, VF-0058–3t, VF-0097–3, and VF-0097–3t were injected with 500 TCID_50_ *D*CV. Each assay was repeated once with males and twice with females with similar results. (D) Fifty 3–6-d-old males, per sample, of VF-0058–3 and VF-0058–3t lines were injected with 50 mM Tris-HCl, pH 7.5, kept at 18°C and their survival was followed daily. (E) Extracts of VF-0058–3 and VF-0058–3t flies 3 and 6 d after injection with 500 TCID_50_ *D*CV or not injected were probed in a Western blot with anti-*D*CV. Anti-tubulin was used as a loading control. (F) Titration, in cell culture, of *D*CV levels per fly of VF-0058–3 and VF-0058–3t flies 3 and 6 d after injection with 500 TCID_50_ *D*CV. Squares are replicates (four per sample), lines are geometric means of replicates. Virus titres in VF-0058–3 and VF-0058–3t are significantly different on both days post-infection (Mann-Whitney test, *p* = 0.0287 for both comparisons).

In the initial screen, we assayed adult survival after intra-thoracic *D*CV injection and realized that, unexpectedly, the control *w^1118^ iso* line was much more sensitive to *D*CV than most of the tested *P*-element insertion lines (Figure 1 and unpublished data). When injected with a dose of 500 times the median tissue culture infective dose (TCID_50_), all the *w^1118^ iso* males died within 12 d, whereas very little death was observed in the males of the *P*-element insertion lines (Figure 1A). Moreover, a large proportion of males of the *P*-element insertion line survived until 21 d after infection. Preliminary analysis showed that the *P*-element was not responsible for virus resistance (unpublished data). We then tested the hypothesis that a previous treatment of the *w^1118^ iso* line with tetracycline could have rendered it more sensitive to *D*CV. We raised flies of a *P*-element insertion line, VF-0058–3, on tetracycline-containing medium or control medium and compared the adults' resistance to *D*CV infection (Figure 1A). The tetracycline treatment made the flies die much faster upon *D*CV infection, with a sensitivity similar to that of *w^1118^ iso* line. This result strongly suggested that a tetracycline-sensitive bacteria, associated with the resistant stocks, conferred resistance to the viral infection. We discarded the possibility that the effect was an artifact of the tetracycline *per se* because raising *w^1118^ iso* flies on medium with tetracycline did not make them more sensitive to *D*CV (Figure 1B). We also treated the VF-0058–3 and VF-0097–3 *P*-element insertion lines with tetracycline for two generations and moved them back to normal medium for at least five generations in order to negate any side effects of tetracycline itself (these stocks will be referred as VF-0058–3t and VF-0097–3t). We then repeated the assay, comparing resistance to *D*CV of these treated stocks to the non-treated stocks (Figure 1C). The tetracycline treatment makes the *P*-element insertion lines stably more sensitive to *D*CV than non-treated lines and equally sensitive to *D*CV as the *w^1118^ iso* line. A similar result was obtained when females of these lines were injected with *D*CV, with the difference that females are less sensitive to *D*CV than males (unpublished data). Importantly, in the timeframe of this assay, the survival of tetracycline-treated and non-treated stocks do not differ when only injected with buffer (Figure 1D). In summary, these results show that tetracycline-sensitive bacteria, not easily acquired from the laboratory environment, confer on D. melanogaster resistance to *D*CV.

The increased resistance to *D*CV could be due to increased resistance to the damage caused by the viral infection or decreased viral proliferation. To test this, we probed by Western blot the levels of viral proteins in VF-0058–3 and VF-0058–3t adult flies after infection with *D*CV (Figure 1E). While viral proteins were not detectable on extracts of *D*CV-infected VF-0058–3 flies, they were clearly detectable on extracts of *D*CV-infected VF-0058–3t flies, and their levels increased from 3 to 6 d post-infection. We extended this analysis by quantifying, in cell culture, the viral titer in these flies after *D*CV infection (Figure 1F). *D*CV is detected after infection in flies from both stocks and slightly increases from 3 to 6 d post-infection. However, *D*CV levels are considerable higher, by approximately 10,000 times, in VF-0058–3t flies. These experiments show that the bacteria that confer resistance to *D*CV infection interfere with the virus proliferation.

### Identification of the Viral Resistance–Inducing Bacteria

**Figure 2:**
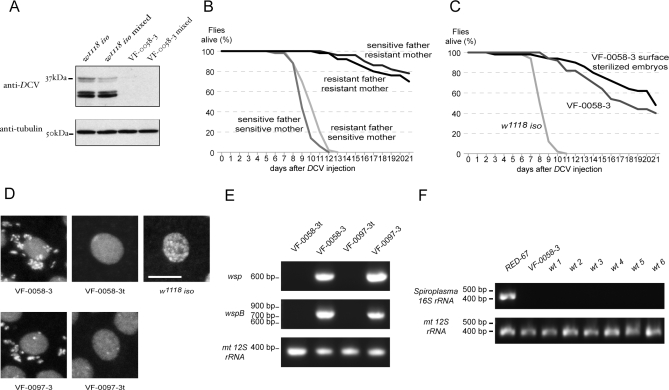
Identification of *Wolbachia* as the Bacteria Inducing *D*CV Resistance (A) Extracts of flies 6 d after injection with 500 TCID_50_ *D*CV were probed in a Western blot with anti-*D*CV. Anti-tubulin was used as a loading control. Flies used were: *w^1118^ iso* ; *w^1118^ iso* raised with VF-0058–3; VF-0058–3; and progeny of VF-0058–3 raised with *w^1118^ iso*. (B and C) Fifty 3–6-d-old males, per sample, were injected with 500 TCID_50_ *D*CV, and their survival followed daily. (B) Males and females from resistant (VF-0058–3) and sensitive (VF-0058–3t) stocks were crossed in the four possible combinations, and their progeny were tested for *D*CV resistance. The assay was repeated with females with similar results. (C) VF-0058–3 embryos were surface sterilized with bleach, raised to adults, and their resistance to *D*CV compared with non-treated VF-0058–3 and *w^1118^ iso* flies. The assay was repeated with females with similar results. (D) DNA staining, with propidium iodide, of 0–2 h embryos of VF-0058–3, VF-0058–3t, *w^1118^ iso*, VF-0097–3, and VF-0097–3t. Extranuclear DNA staining corresponds to bacteria. Scale bar, 10μm. (E) PCR amplification with *wsp* and *wspB* primers on DNA extracts of VF-0058–3t, VF-0058–3, VF-0097–3t, and VF-0097–3 embryos. PCR amplification with *mt 12S rRNA* primers was done as a DNA extraction control. (F) PCR amplification with primers specific for *Spiroplasma 16S rRNA* gene on DNA extracts of *RED-67*, *VF-0058–3*, *wt 1*, *wt 2*, *wt 3*, *wt 4*, *wt 5*, and *wt 6* adults. PCR amplification with *mt 12S rRNA* primers was done as a DNA extraction control.

The fact that the tetracycline treatment permanently renders the flies sensitive to *D*CV shows that the bacteria are not easily acquired from the laboratory environment. To test if the resistance could be horizontally acquired, we raised together the progeny of *w^1118^ iso* females (without resistant-inducing bacteria) with the progeny of VF-0058–3 females (with resistant-inducing bacteria) and then assayed the levels of viral proteins in infected flies (Figure 2A). The progeny of these females can be distinguished by their eye color, due to the presence of a functional *white* gene, in the *RS3* transposon, only in the progeny of VF-0058–3 flies. *w^1118^ iso* flies do not acquire the resistance to *D*CV when raised mixed with the progeny of *VF-0058–3* flies. Therefore, the bacteria that confer resistance to *D*CV are not acquired horizontally. We then tested if the resistance was vertically transmitted by crossing males and females from VF-0058–3 and VF-0058–3t stocks in all four possible combinations and assaying the survival of the adult progeny after *D*CV infection (Figure 2B). The results clearly show that the determinant factor of the progeny resistance is the mother's resistance; therefore, the bacteria in question are maternally transmitted, which strongly suggests they are intracellular. However, the bacteria could, in theory, be only transmitted by the mother but not be intracellular (e.g., they could be deposited on the egg surface). This did not seem to be the case, because flies that were raised from VF-0058–3 surface-sterilized embryos did not become more sensitive to *D*CV (Figure 2C). Moreover, we could visualize the presence of intracellular bacteria by DNA staining in embryos from VF-0058–3 and VF-0097–3 stocks but not from VF-0058–3t, VF-0097–3t, or *w^1118^ iso* stocks (Figure 2D). We can therefore conclude that the viral resistance is mediated through maternally transmitted intracellular bacteria.

**Figure 3:**
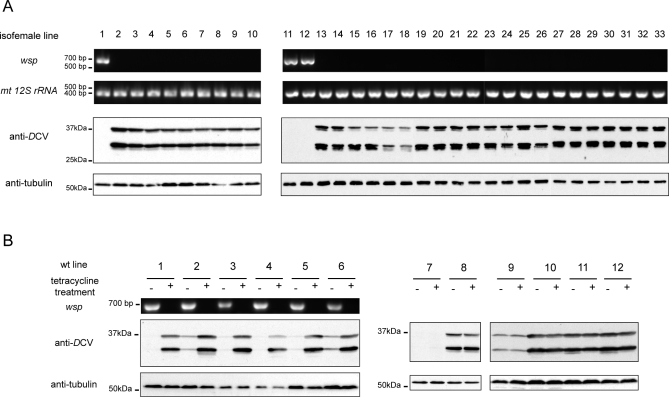
Confirmation of *Wolbachia* as the Bacteria Inducing *D*CV Resistance (A) Two independent sets of isofemales lines were established from VF-0058–3 flies raised on a sub-optimal dose of tetracycline (lines 1–10 and 11–33). The presence of *Wolbachia* in these lines was tested by PCR amplification using *wsp* primers on DNA extracts of adult flies, PCR with *mt 12S rRNA* primers was done as a control. Three–six-d-old males of each line were injected with 500 TCID_50_ *D*CV, collected 6 d later, and *D*CV levels analysed by Western blot with anti-*D*CV. Anti-tubulin was used as a loading control. (B) Six wild-type lines infected with *Wolbachia* (lines 1–6) and six wild-type lines not infected (lines 7–12) were identified by PCR amplification with *wsp* primers (more information on wild-type lines identity can be found in Materials and Methods). Each stock was treated with tetracycline for two generations and then transferred to tetracycline-free food for at least two generations. Treated and non-treated stocks were re-tested for presence of *Wolbachia* by PCR amplification with *wsp* primers. Seven–twelve PCR amplifications were all negative, not shown. Three–six-d-old males of each stock (lines 1–12, tetracycline treated and non-treated) were injected with 500 TCID_50_ *D*CV, collected 6 d later, and *D*CV levels analysed by Western blot with anti-*D*CV. Anti-tubulin was used as a loading control.

To identify the intracellular bacteria in question, we extracted DNA from surface-sterilized embryos of resistant-to-*D*CV flies (VF-0058–3) and performed PCR amplification using prokaryotic *16S rRNA* universal primers. We analysed the product of this amplification by cloning it and sequencing over 100 independent clones. All the 104 sequences of inserts in the cloning plasmid we obtained were at least 99.5% identical to the sequence of a fragment of the *16S rRNA* gene of *Wolbachia* (GenBank accession number EU096232; http://www.ncbi.nlm.nih.gov/Genbank/). Therefore, these embryos, which carry the resistance to *D*CV inducing bacteria, are most probably only infected with *Wolbachia*. To verify the presence of *Wolbachia*, we performed PCR amplification using primers for the *Wolbachia* specific genes *wsp* and *wspB* [45,46] (Figure 2E). *Wolbachia* is present in VF-0058–3 and VF-0097–3 and absent from the VF-0058–3t and VF-0097–3t embryos' extracts. The sequence of the *wsp-*specific primers' PCR amplification product from the VF-0058–3 flies is identical to the *wsp* sequence of *wMe*l (GenBank accession number DQ235407), the only *Wolbachia* strain known to infect D. melanogaster. In a recent survey in 35 different *Drosophila* species, that screened over 4,500 individuals, only two kind of heritable endosymbiotic bacteria were found: *Wolbachia* and *Spiroplasma* [47]. We specifically tested for the presence of *Spiroplasma* in the VF-0058–3 line (and the *wt-1* to -*6* lines used in Figure 3B) using primers specific for the *16S rRNA* gene of *Spiroplasma* [48]. We detect *Spiroplasma* in a positive control, RED-67 [48], but not in any of the other tested lines. Therefore it is not *Spiroplasma* that confers resistance to viruses. This result and the sequencing results strongly suggest that the maternally inherited intracellular bacteria that confer resistance to *D*CV are *Wolbachia*.

These results show that resistance to *D*CV is associated with the presence of *Wolbachia*, however it could be possible that other cryptic intracellular bacteria were responsible for the virus resistance, and *Wolbachia* would merely be present in these flies by chance. *Wolbachia* cannot be cultured and therefore we cannot infect a sensitive stock with a pure cultured isolate and verify acquired resistance to *D*CV. *Wolbachia* can be artificially transferred from an infected host to a new host. However, if we did transfer *Wolbachia* from infected flies to non-infected flies and show concomitant transfer of resistance to *D*CV, we could not discard the possibility that we were also transferring the hypothetical cryptic bacteria. We addressed this problem by treating the *Wolbachia-*infected stock VF-0058–3 with a suboptimal dose of tetracycline for one generation and then establishing isofemale lines from the progeny. We expected to obtain lines that kept the *Wolbachia* infection and other lines that lost it. The segregation of *Wolbachia* should be independent of the segregation of any hypothetical other bacteria. From two independent sets, one set of ten lines and another set of 23 lines, we established, in total, three lines that conserved *Wolbachia* infection and 30 lines that lost it. We then tested these lines for resistance to viruses (Figure 3A). *Wolbachia* presence and viral resistance fully segregate with each other; the probability that the presence of *Wolbachia* and resistance to *D*CV are independent is very low (Fisher's exact test, *p* = 0.0002). These data strongly indicate that it is *Wolbachia* infection that induces *D*CV resistance.

To corroborate that it is *Wolbachia* infection that protects D. melanogaster from *D*CV, we analyzed this interaction in other independent fly stocks. We screened, by PCR, for *Wolbachia* presence in a collection of wild-type stocks kept in our laboratory and we found six infected lines. After establishing tetracycline-treated stocks derived from these lines, we compared, by Western blots, their resistance to *D*CV with the original lines (Figure 3B). In all the six cases, the loss of *Wolbachia* makes the flies more sensitive to *D*CV. The same procedure was applied to six stocks that did not carry *Wolbachia* initially (Figure 3B). There is much heterogeneity in the levels of *D*CV proteins 6 d after infection in these stocks (e.g., line 7 is very resistant to *D*CV infection), but, importantly, there is no increase in the levels of *D*CV proteins in the tetracycline treated stocks. These results show that increase sensitive to *D*CV after tetracycline treatment is always associated with an initial *Wolbachia* infection. From this set of data, plus the same result of increased sensitivity with tetracycline treatment of VF-0058–3, we can state that the probability that initial *Wolbachia* infection and increased sensitivity upon tetracycline treatment are independent is very low (Fisher's exact test, *p* = 0.0006). In conclusion, we can confidently state that it is *Wolbachia* that protects D. melanogaster from *D*CV infection.

### *Wolbachia* Effect on Other Viral Infections

*Wolbachia* and *D*CV are commonly found in D. melanogaster natural populations and laboratory stocks; their interaction could be very specific. We investigated if *Wolbachia* protection extends to infections by two other RNA viruses and a DNA virus.

**Figure 4:**
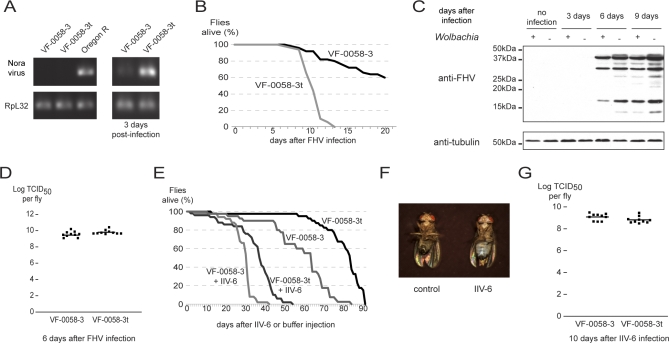
*Wolbachia* Interaction with Other Viruses (A) RT-PCR was done with Nora virus primers on RNA of VF-0058–3, VF-0058–3t, and Oregon R flies (left). PCR with *RpL32* was done as control. Three–six-d-old males of VF-0058–3 and VF-0058–3t lines were injected with a virus extract of Oregon R flies and collected 3 d later. RNA was extracted and RT-PCR done with primers for Nora virus and *RpL32.* The same number of PCR cycles was done for both samples. The assay was repeated three more times, from infection of flies with virus extract, with similar results. (B) Fifty 3–6-d-old males, per sample, of VF-0058–3 and VF-0058–3t lines were injected with 50 TCID_50_ FHV, and their survival was followed daily. The assay was repeated twice with males and once with females with similar results. (C) Extracts of VF-0058–3 and VF-0058–3t flies 3, 6, and 9 d after injection with 50 TCID_50_ FHV or not injected were probed in a Western blot with anti-FHV. Anti-tubulin was used as a loading control. (D) Titration, in cell culture, of FHV levels per fly of VF-0058–3 and VF-0058–3t flies 6 d after injection with 50 TCID_50_ FHV. Squares are replicates (10 per sample), lines are geometric means of replicates. Virus titres in VF-0058–3 and VF-0058–3t are not significantly different (Mann-Whitney test, *p* = 0.05764). (E) Fifty 3–6-d-old males, per sample, of VF-0058–3 and VF-0058–3t lines were injected with 1,000 TCID_50_ IIV-6 or buffer, and their survival followed. The assay was repeated once with males and the IIV-6 injected flies survival curves were also repeated with females, with similar results. (F) Iridescent-infected male 20 d after injection with 1000 TCID_50_ IIV-6 (right) and not infected same age male (left) are shown. (G) Titration, in cell culture, of IIV-6 levels per fly of VF-0058–3 and VF-0058–3t flies 10 days after injection with 1000 TCID_50_ IIV-6. Squares are replicates (10 per sample), lines are geometric means of replicates. Virus titres in VF-0058–3 and VF-0058–3t are not significantly different (Mann-Whitney test, *p* = 0.08118).

Nora virus is a recently described common natural pathogen of D. melanogaster [49]. Similar to *D*CV, Nora virus is a small, non-enveloped virus with a single-stranded, positive-sense RNA genome. It is similar to picornaviruses and dicistroviruses, but it has a unique genome organization. Using reverse-transcription PCR (RT-PCR) with Nora virus–specific primers, we found that the VF-0058–3 and VF-0058–3t stocks are not infected with it while another laboratory stock, Oregon R, is (Figure 4A). An extract of Oregon R adult flies was injected into VF-0058–3 and VF-0058–3t adult flies, and the levels of Nora virus replication were accessed, by semi-quantitative RT-PCR, after 3 d (Figure 4A). We observed, in four independent repeats, that Nora virus levels are lower in *Wolbachia-*infected flies; *Wolbachia* infection also protects *Drosophila* from Nora virus infection.

Flock House virus (FHV) belongs to the Nodaviridae family of insect viruses. These are small viruses containing two single-stranded, positive-sense genomic RNAs [50]. FHV was isolated from a coleopteran [51] and is not a natural pathogen of D. melanogaster. However, it can be cultured in D. melanogaster cells and proliferates and causes death in adult flies when injected [38,50]. We injected FHV in VF-0058–3 and VF-0058–3t adult flies and followed their survival (Figure 4B). *Wolbachia-*infected flies were much more resistant to FHV infection; with an infection dose of 50 TCID_50_, all VF-0058–3t flies die by day 13, while only 40% of VF-0058–3 flies die by day 21. We can detect, by Western blot, increase in FHV proteins with time in both infected stocks (Figure 4C). Surprisingly, we only detected a slight increase in FHV proteins in the infected VF-0058–3t flies, compared with the infected VF-0058–3 flies. We confirmed this result by determining, in cell culture, the viral titer per infected fly, 6 d post-infection (Figure 4D). We find, on average, only 1.8-fold more FHV in *Wolbachia-*free flies. and the difference is not statistically significant (Mann-Whitney test, *p* = 0.05764). We can conclude that *Wolbachia* presence also increases the resistance of D. melanogaster to FHV infection although it does not or only slightly affects FHV levels.

Finally, we wanted to test the effect of *Wolbachia* on a DNA virus infection; however, there is no known DNA virus that is a natural pathogen of D. melanogaster. Insect Iridescent Virus 6 (IIV-6) (also named *Chilo* iridescent virus (*C*IV)) is a large virus with a double-stranded DNA genome from the Iridoviridae family [52]. It was first isolated from a lepidopteran but can infect a large number of different insects and cultured insect cells [53,54]. IIV-6 can infect and replicate in D. melanogaster cells [54] and cause adult flies death upon injection (Peter Christian, personal communication). We confirmed that IIV-6 infection causes premature death in adult flies, approximately halving their lifespan when 1,000 TCID_50_ are injected per fly (Figure 4E). Importantly we can show that IIV-6 replicates in adult D. melanogaster. Infected flies become iridescent as they accumulate virions, a characteristic of iridoviruses due to their paracrystalline packing (Figure 4F). Moreover, IIV-6 titer per fly after 10 d of infection, determined in cell culture, was approximately 10^9^ TCID_50_, when a dose of 10^3^ TCID_50_ was injected (Figure 4G). Contrary to the results obtained with *D*CV and FHV infection, *Wolbachia-*infected flies actually died faster than *Wolbachia*-free flies when infected with IIV-6 (Figure 4E). This probably represents just a cumulative effect of the deleterious effects of *Wolbachia* and IIV-6 infection. In fact, *Wolbachia* infection has a long-term deleterious effect that results in a shorter lifespan in the absence of viral infection (Figure 4E). In accordance with this interpretation, the average IIV-6 titer, 10 d after injection, is only 1.8-fold higher in *Wolbachia-*infected flies compared with *Wolbachia*-free flies, and not significantly different (Mann-Whitney test, *p*=0.08118) (Figure 4G). In conclusion, *Wolbachia* presence does not protect D. melanogaster from IIV-6 infection.

## Discussion

We have shown that *Wolbachia* infection in *D. melanogaster* induces resistance to *D*CV infection. Several lines of evidence lead to this conclusion. The resistance to *D*CV was maternally transmitted and sensitive to tetracycline, as is *Wolbachia*; in embryos infected with bacteria inducing resistance to *D*CV, we can only detect the presence of *Wolbachia*; all tested D. melanogaster lines that carried *Wolbachia* became more sensitive to *D*CV after tetracycline treatment; lines that did not carry *Wolbachia* did not become more sensitive to *D*CV after tetracycline treatment. Finally, when transmission to the next generation was imperfect, due to treatment of larvae with a low dose of tetracycline, *Wolbachia* and resistance to *D*CV co-segregated. Following Occam's razor principle—*Pluralitas non est ponenda sine necessitate. “*Plurality should not be posited without necessity.” —the simplest and most plausible hypothesis is that *Wolbachia* is the causative agent of resistance to *D*CV.

Infection by *Wolbachia* considerably increased the lifespan of *D*CV-infected flies. This is due to a strong reduction in viral titers, as observed by Western blot and titration by cell culture. At 3 d post infection, the *D*CV titer in *Wolbachia-*infected flies was 10,000 times less than that in *Wolbachia* free flies. This difference is larger than that reported between the wild type and mutants in the anti-viral resistance genes *Dcr-2*, *ago-2*, and *hop* [36,38,40]. *Wolbachia* is clearly a major factor affecting *Drosophila* resistance to *D*CV.

*Wolbachia* and *D*CV are common symbionts of *D. melanogaster.* However, the interaction is not specific to *D*CV; we found that *Wolbachia* also induced resistance to two other RNA viruses. In the case of Nora virus, there was also reduction in the viral titer of infected flies. FHV infection, in terms of mortality, was also much less severe in the presence of *Wolbachia*, to a degree similar to that seen with *D*CV. But, with this virus, *Wolbachia* only slightly affected viral titer. The resistance to FHV is most probably an increase in resistance to the damaged caused by the viral infection rather than an ability to inhibit virus proliferation. However, we cannot exclude the possibility that there is strong inhibition of FHV proliferation in certain essential adult tissues or that a small decrease in viral titer is enough to significantly increase the lifespan of infected individuals.

*D*CV and Nora virus differ from FHV in two ways: they are both natural pathogens of *Drosophila* and both are picornavirus-like. An endogenous virus and its host could be co-adapted so that a small advantage, in this case provided by the bacteria to the host, would profoundly tilt the equilibrium between virus and host, whereas an exogenous pathogen may be less sensitive to bacterial infection of its host. On the other hand, *Wolbachia* could interfere with the life cycle of picornavirus-like viruses but not of FHV, a nodavirus. We cannot distinguish between these possibilities with such a small sample of viruses; it would be interesting to extend the analysis to other RNA viruses that infect D. melanogaster (e.g., Sigma (a rhabdovirus) [43], *Drosophila* X virus (a birnavirus) [55], and *Drosophila* A virus (picornavirus-like) [43]).

We have also tested the interaction of *Wolbachia* with a DNA virus, IVV-6. *Wolbachi*a did not protect *Drosophila* from this virus; it actually decreased the lifespan of infected flies. We think this is due to the cumulative effect of *Wolbachia* and IIV-6 infection, since, in the genetic background of the flies we were using, *Wolbachia* had a negative effect on long-term survival. It would be interesting to also extend the analysis to other DNA viruses, however there are no DNA viruses known to infect D. melanogaster. To our knowledge, this is the first report of a DNA virus proliferating in adults of *D. melanogaster.*

An obvious question is how *Wolbachia* induces resistance to RNA viruses. The different effect on *D*CV/Nora virus and FHV raises the possibility that this effect is multifactorial; interfering with virus replication in some cases and increasing resistance of *Drosophila* to viral infection damage in others. One important question to address is whether the effect is cell-autonomous or systemic. *Wolbachia* is widespread throughout tissues of the infect host [18,56], so both hypotheses are possible. This could be investigated in tissue culture with *Wolbachia-*infected cells. If the effects are cell autonomous, one explanation for increased resistance to viruses could just be competition for resources, since both microorganisms occupy the same niche, the host's cytoplasm. For example, *Wolbachia* is thought to acquire much of its energy from the metabolism of amino acids imported from the host cytoplasm [46]. *D*CV, on the other hand, is very sensitive to perturbations in host translation [57]. The presence of *Wolbachia* could reduce the pool of cytoplasmic amino acids to a point that interferes with translation of viral proteins. Another possibility is that *Wolbachia* infection could trigger cell-autonomous mechanisms of resistance to intracellular pathogens, such as a reduction in cellular metabolism. A further explanation for a cell-autonomous effect would be that *Wolbachia* has been selected to actively interfere with virus replication in co-infected cells. *Wolbachia* has a complete type IV secretion system [46], which many bacteria use for translocation of effector molecules into host cells (e.g., *Legionella* and *Agrobacterium*). Genes encoding proteins with ankyrin repeats, involved in protein–protein interactions, are over-represented in the *Wolbachia* genome [46,58] and are good candidates for mediators of anti-viral resistance.

If the effect is systemic, a strong hypothesis is that *Wolbachia* could alter the host–immune response, increasing resistance to viral infection. The pre-activation of the host immune system, for example, could allow for a faster response upon viral infection. This would be similar to what happens in a herpesvirus-induced resistance to *Listeria* in mice, due to the production of cytokines [59]. It was also reported recently that the presence of gut flora slightly increases the resistance of Aedes aegypti to Dengue virus, presumably through activation of the Toll pathway [60]. In tissue culture of D. melanogaster cells, infection with *Wolbachia* slightly increases the expression of innate immune genes [61]. There is also a report that *Wolbachia* increases resistance of D. melanogaster to the pathogenic fungus Beauveria bassiana [62]. All these reports support a model of general activation of innate immunity. However it has also been shown that in adult D. simulans and *Aedes albopictus Wolbachia* does not activate the expression of anti-microbial peptides [63], in D. simulans, *Wolbachia* infection does not alter sensitivity to *Beauveria* and renders the host more sensitive to parasitoid wasps [64], and in D. melanogaster, *Wolbachia* presence does not affect *Spiroplasma* levels [65]. In summary, it is not clear if there is a general activation of innate immunity in adult D. melanogaster infected with *Wolbachia* that would render them more resistant to other pathogens. It would be interesting to identify immune pathways involved in anti-viral resistance activated by *Wolbachia* infection. It would also be important to analyze *Wolbachia-*induced resistant to other microorganisms, including pathogenic bacteria.

A different hypothesis would be that *Wolbachia* infection actually inhibits some of the immune responses against viral infection and that increases the lifespan of infected *D. melanogaster.* This may be true if the host response to infection damages the host itself, as in the case of septic shock in mammals. This could explain the increased resistance to FHV infection without a strong effect on viral titers. Finally, a similar hypothesis would be that *Wolbachia* inhibits, cell-autonomously or systemically, apoptosis induced upon viral infection. Some published data support this hypothesis; FHV induces apoptosis in tissue culture cells [66], *Wolbachia* inhibits apoptosis in the germline of Asobara tabida [8,9], and the *Wolbachia* protein Wsp inhibits apoptosis in human cells [67].

This new host-microorganism-microorganism interaction adds to the perception that the response of a host to a particular pathogen also depends on its interactions with other microorganisms. Other examples are herpesvirus latency-induced protection to *Listeria* in mice mentioned above [59], the suppression of HIV-1 infection by human herpesvirus 6 in human cells [68], symbiotic bacteria protection against fungi in a shrimp and an aphid [69,70], symbiotic bacteria protection against parasitic wasps in an aphid [71], and symbiotic bacteria protection against fungal infection in a wasp [72]. As also mentioned above, there is a recent report that gut flora has a protective role against Dengue virus in A. aegypti [60]. However, this is, to our knowledge, the first report where bacteria that confer protection against viruses have been identified.

This interaction has some practical consequences. Researchers working on *Drosophila* immunity against viruses should take in consideration the presence of *Wolbachia* in the stocks they are analyzing. On the other hand, researchers working on *Wolbachia* should consider that any observed effects of *Wolbachia* could be mediated through effects on viral infections. A practical application of this discovery would be, if possible, to induce resistance to viruses, by infection with *Wolbachia,* in insects that are beneficial to humans (e.g., honeybee) or transmit arboviruses (e.g., mosquitoes). However, introducing *Wolbachia* to virus-transmitting vectors could be a double-edged sword. If the interaction *Wolbachia*-vector-virus were similar to the one seen in this report with *D*CV, then it would be beneficial because it could decrease the probability of the vector being infected or transmitting the disease. If, however, it were similar to the interaction with FHV, then there would be the risk of having healthier infected vectors with high titers of viruses, therefore increasing disease transmission. This latest possibility should be taken into account in proposed strategies of introducing *Wolbachia* in vectors of arboviruses [73,74].

Finally, this is, to our knowledge, the first report of a strong beneficial effect of *Wolbachia* infection in D. melanogaster. The induced resistance to natural viral pathogens may explain the prevalence of *Wolbachia* in natural populations. It also indicates that the fitness benefit of having *Wolbachia* is dependent on the viral infection status of the population. This may explain differences in *Wolbachia* infection frequencies between populations [17,25] and variable fitness effects in different D. melanogaster lines [28,30]. It would be interesting to broaden the analysis to other *Wolbachia* strains and to other *Wolbachia–*host combinations. If *Wolbachia* induces resistance to viruses in other hosts, this would have major implications for our understanding of the very widespread presence of this endosymbionts in arthropods and filarial nematodes.

After the submission of this manuscript, an independent report with similar findings to ours was published [75]. In agreement with our data, Hedges et al. show that the treatment of *Wolbachia-*infected flies with tetracycline renders them more sensitive to three RNA viruses: *D*CV, Cricket Paralysis virus, and FHV. Moreover, they also show that the levels of *D*CV increase in infected *Wolbachia*-free flies.

## Materials and methods

### Fly strains and husbandry.

The set of *w^1118^ iso* isogenic flies were obtained from the DrosDel collection in our laboratory [42]. These lines were cleaned of viruses similarly to the protocol in Brun and Plus, 1978 [43]. Flies were aged to 30 d at 25 °C and their eggs were collected in agar plates, treated with 50% bleach for 10 min, washed with water, and transferred to fresh vials.

The wild-type laboratory lines used in Figure 3B have the origins described in Table 1.

**Table 1:**
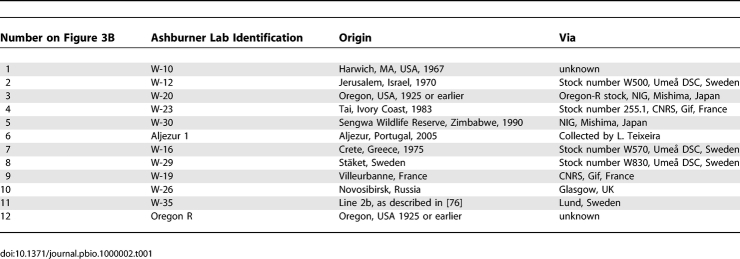
Origins of Wild-Type Laboratory Lines Used in This Work

Stocks were treated with tetracycline (cleaned of *Wolbachia* infection) by raising them for two generations in ready-mix dried food (Philip Harris) with 0.05 mg/ml of tetracycline hydrochloride (Sigma). Sub-optimal tetracycline treatment was done by raising flies, for one generation, in food with 0.00625 mg/ml of tetracycline hydrochloride. Virgin adult females, emerging from these vials, were collected and individually crossed with males to establish isofemale lines.

Sensitive and resistant-to-viruses flies were raised together by placing in a vial one *w^1118^ iso* female, one *VF-0058–3* female, and two *w^1118^ iso* males. The progeny of the two different females could be distinguished by the eye color, because only the progeny of *VF-0058–3* females have a functional *white* gene. Flies were only collected from vials that had adults of both phenotypes.

### Virus production.

*D*CV-C [77] was kindly provided by Dr. Peter Christian and raised as in Johnson and Christian 1999 [78].

Nora virus extract was prepared from a naturally infected Oregon stock present in the laboratory. Thirty adult flies were squashed in 900 μl of 50 mM Tris-HCl, pH 7.5. Extract was then frozen at −80 °C, thawed and twice centrifuged for 10 min at top speed in a tabletop centrifuge, at 4 °C. The supernatant was aliquoted and stored at −80 °C.

IIV-6 [79] was kindly given by Dr. Peter Christian and raised in Schneider *Drosophila* line 2 (DL2) cells [54]. DL2 cells were kept in Schneider's *Drosophila* Medium (Invitrogen) supplemented with 10% Fetal Bovine Serum, 2mM L-Glutamine, 100 U/ml penicillin, and 100μg/ml streptomycin (all Invitrogen). Seven days after infection, the cell culture was collected and frozen at −80 °C. The culture was thawed, frozen, and thawed again to disrupt cells and centrifuged twice at 600*g* for 10 min to remove cell debris. Virus was pelleted by centrifugation at 10,000*g* for 10 min and re-suspended in water. Virus suspension was placed over a 30% sucrose solution and the virus was pelleted again by centrifugation at 30,000*g* for 30 min and re-suspended in water. The virus was then pellet by centrifugation at 15,000*g* for 10 min and re-suspended twice. The final re-suspension was done in 50 mM Tris-HCl, pH 7.5, aliquoted, and stored at −80 °C. All centrifugations and re-suspensions were done at 4 °C.

FHV was kindly provided by Dr. J.-L. Imler [38] and dilutions of this aliquot were used in this work.

### Virus injection.

Three-to-six–d-old flies were injected with a Nanoject II injector (Drummond). Viruses were re-suspended or diluted in 50 mM Tris-HCl, pH 7.5, and 69 nl of virus solution was injected, per fly, in the thorax, between the mesopleura and the pteropleura. Flies were injected while anesthetized with CO_2_. Fifty flies were injected per sample, ten flies were placed per vial, and vials were changed twice a week. *D*CV injected flies were kept at 18 °C. Nora virus–, FHV- and IIV-6–injected flies were kept at 25 °C. Flies were counted daily for all survival curves except for VF-0058–3 and VF-0058–3t injected with buffer, as shown in Figure 4E, which were counted at least twice a week.

### Virus titration.

Five flies were pooled per sample. Flies were squashed in 50 mM Tris-HCl, pH 7.5, frozen, thawed, and centrifuged for 10 min at 20,000*g* and supernatant was collected (DCV and FHV). For IIV-6, centrifugation was done twice at 600*g* and the supernatant passed through a 0.45-μm filter before the assay. Viruses titers were determined in cell culture and calculated by the Reed and Muench end-point calculation method [80]. *D*L2 cells in 96-well plates were infected with the serial dilutions of virus suspensions. *D*CV and FHV infection was scored by the presence of cell death, IIV-6 was scored by non-proliferation of cells and presence of very large cells. Extracts of non-infected VF-0058–3 or VF-0058–3t flies did not cause any cytopathic effect in tissue culture cells.

### Western blots.

Five to eight males were pooled per sample. Rabbit polyclonal antibodies raised against purified *D*CV was kindly given by Dr. Peter Christian. Rabbit polyclonal antibodies raised against FHV capsids was kindly given by Dr. Jean-Luc Imler [38]. Specificity of antibodies was verified by lack of signal on Western blot lanes of non-infected flies (Figures 1E and 4C). E7 mouse monoclonal anti-β-tubulin was acquired from Developmental Studies Hybridoma Bank [81].

### Propidium iodide staining.

Embryos 0–2-h-old were collected, treated with 50% commercial bleach for 10 min, fixed for 30 min in 4% formaldehyde, 50% heptane, the vitelline membranes were removed by vortexing the embryos in 50% heptane, 50% methanol, then embryos were washed briefly in methanol and for 10 min in 50% methanol, 50% PBS and finally placed in PBS 0.1% tween-20. Embryos were treated with RNAse H 0.25 μg/μl for 30 min at 37 °C, washed in PBS 0.1% tween-20, stained with PBS 0.1% tween-20 and 1 μg/ml propidium iodide (Sigma) for 30 min, washed in PBS 0.1% tween-20 and mounted in Vectorshield. Images were taken in a confocal microscope.

### PCR, sequence analysis, and RT-PCR.

A fragment of bacterial *16S rRNA* gene was amplified from DNA of *Drosophila* embryos surface sterilized by treatment with 50% commercial bleach for 10 min. DNA was extracted using Wizard Genomic DNA purification kit (Promega). Primers used were 27f (5′-GAGAGTTTGATCCTGGCTCAG-3′) and 1495r (5′-CTACGGCTACCTTGTTACGA - 3′). The PCR program was: 94 °C for 4 min; 25 cycles of 94 °C for 30 s, 58 °C for 1 min, and 72 °C for 2 min; 72 °C for 10 min. The PCR product was ligated into pCR 2.1 TOPO vector (Invitrogen) and transformed into DH5α cells. Nineteen plasmid DNA preparations and 96 bacteria cultures were sent for sequencing. Three sequencing reactions failed and 8 clones did not carry an insertion in the cloning plasmid. From all the other 104 sequences, we selected a sequence of at least 600 bp with good quality and aligned it with a fragment of the *16S rRNA* gene of *Wolbachia* (GenBank accession number EU096232) using Clustal W 2 [82].

PCR amplification of *Wolbachia-*specific genes was done either on the DNA extracts of embryos as described above (Figure 2E) or on DNA extracts of adult flies (Figure 3). Adult flies were squashed in 25 mM NaCl, 10 mM Tris-HCl pH=8.0, 1 mM EDTA, 200 μg/ml proteinase K and incubated for 30 min at 37 °C. Proteinase K was inactivated at 95 °C for 5 min. The supernatant was directly used for PCR amplification. *wsp* primers were *wsp* 81F (5′-TGGTCCAATAAGTGATGAAGAAAC-3′) and *wsp* 691R (5′-AAAAATTAAACGCTACTCCA-3′) [45]. *wspB* primers were *wspB*-F (5′-TTTGCAAGTGAAACAGAAGG-3′) and *wspB*-R (5′-GCTTTGCTGGCAAAATGG-3′) [46]. As a positive control for cytoplasmic DNA extraction we used the primers for mitochondrial *12S rRNA*, 12SAI (5′-AAACTAGGATTAGATACCCTATTAT-3′) and 12SBI (5′-AAGAGCGACGGGCGATGTGT-3′) [4]. The PCR program used was: 94 °C for 4 min; 30 cycles of 94 °C for 1 min, 55 °C for 1 min, and 72 °C for 1 min; 72 °C for 10 min. The *wsp* primers amplification product from VF-0058–3 embryos was purified, as described above, and sequenced. The sequence obtained was identical to a fragment of the *wsp* sequence of wMel (GenBank accession number DQ235407).

PCR amplification with primers specific for Spiroplasma *16S rRNA* gene was done on DNA extracts of adult flies. Primers used were SpoulF (5′-GCTTAACTCCAGTTCGCC-3′) and SpoulR (5′-CCTGTCTCAATGTTAACCTC-3′) [48]. The PCR program was: 94 °C for 4 min; 30 cycles of 94 °C for 30 s, 55 °C for 1 min, and 72 °C for 1 min; 72 °C for 10 min. As a positive control for cytoplasmic DNA extraction, we used the primers for mitochondrial *12S rRNA*, as described before.

Nora virus presence in VF-0058–3, VF-0058–3t, and Oregon R stocks was analyzed by RT-PCR. RNA of 100 flies, per sample, was extracted using Trizol (Invitrogen). cDNA was synthesized from 5 μg of total RNA with Superscript III (Invitrogen), using random hexamers primers, at 50 °C. The Nora primers used for PCR were Nora-F (5′-TTAAGGTGTTAGAGAACAGC-3′) and Nora-R (5′-CGTAAACACCAACTTACTTC-3′) [49]. *RpL32* primers, used as a positive control for RNA extraction, were RpL32-F (5′-TCCTAC CAGCTTCAAGATGAC-3′) and RpL32-R (5′-CACGTTGTGCACCAGGAACT-3′). The PCR program used was as follow: 94 °C for 4 min; 10 cycles of 94 °C for 30 s, 60 °C minus 0.6 °C per cycle for 1 min and 72 °C for 1 min; 20 cycles of 94 °C for 30 s, 54 °C for 1 min, and 72 °C for 1 min; 72 °C for 10 min. The PCR amplification fragment obtained with the Nora primers was purified, as described above, and sequenced. The sequence was 98% identical to a fragment of Nora virus genome sequence (GenBank accession number DQ321720). For semi-quantitative analysis of Nora virus in infected flies, the procedure was as above except that 25 flies were used per sample and the PCR amplification for each sample was done with a total of 20, 25, and 30 cycles.

## Abbreviations

CI: cytoplasmic incompatibility
*D*CV: *Drosophila* C virus
FHV: Flock House virus
IIV-6: Insect Iridescent Virus 6
RT-PCR: reverse-transcription PCR
TCID_50_: median tissue culture infective dose
